# Early Changes in Alpha-Fetoprotein and Des-γ-Carboxy Prothrombin Are Useful Predictors of Antitumor Response to Durvalumab Plus Tremelimumab Therapy for Advanced Hepatocellular Carcinoma

**DOI:** 10.3390/curroncol31080315

**Published:** 2024-07-26

**Authors:** Teiji Kuzuya, Naoto Kawabe, Hisanori Muto, Yuryo Wada, Gakushi Komura, Takuji Nakano, Hiroyuki Tanaka, Kazunori Nakaoka, Eizaburo Ohno, Kohei Funasaka, Mitsuo Nagasaka, Ryoji Miyahara, Yoshiki Hirooka

**Affiliations:** Department of Gastroenterology and Hepatology, Fujita Health University, 1-98 Dengakugakubo, Kutsukake-Cho, Toyoake 470-1192, Japan; kawabe@fujita-hu.ac.jp (N.K.); hisanori.muto@fujita-hu.ac.jp (H.M.); warbleman@gmail.com (Y.W.); wheeze0128@gmail.com (G.K.); tkjnkn@fujita-hu.ac.jp (T.N.); hiroyuki.tanaka@fujita-hu.ac.jp (H.T.); knakaoka@fujita-hu.ac.jp (K.N.); eizaburo.ono@fujita-hu.ac.jp (E.O.); k-funa@fujita-hu.ac.jp (K.F.); nmitsu@fujita-hu.ac.jp (M.N.); ryoji.miyahara@fujita-hu.ac.jp (R.M.); yoshiki.hirooka@fujita-hu.ac.jp (Y.H.)

**Keywords:** hepatocellular carcinoma, durvalumab, tremelimumab, antitumor response, alpha-fetoprotein, des-γ-carboxy prothrombin

## Abstract

The relationship between antitumor response and tumor marker changes was evaluated in patients with advanced hepatocellular carcinoma treated with durvalumab plus tremelimumab (Dur/Tre). Forty patients were enrolled in this retrospective evaluation of treatment outcomes. According to the Response Evaluation Criteria for Solid Tumors version 1.1 at 8 weeks, the objective response (OR) rate was 25% and the disease control (DC) rate was 57.5%. The median alpha-fetoprotein (AFP) ratio at 4 weeks was 0.39 in patients who achieved OR at 8 weeks (8W-OR group), significantly lower than the 1.08 in the non-8W-OR group (*p* = 0.0068); however, it was 1.22 in patients who did not achieve DC at 8 weeks (non-8W-DC group), significantly higher than the 0.53 in the 8W-DC group (*p* = 0.0006). Similarly, the median des-γ-carboxy-prothrombin (DCP) ratio at 4 weeks was 0.15 in the 8W-OR group, significantly lower than the 1.46 in the non-8W-OR group (*p* < 0.0001); however, it was 1.23 in the non-8W-DC group, significantly higher than the 0.49 in the 8W-DC group (*p* = 0.0215). Early changes in tumor markers after Dur/Tre initiation were associated with antitumor response. In particular, changes in AFP and DCP at 4 weeks may offer useful biomarkers for early prediction of both response and progressive disease following Dur/Tre.

## 1. Introduction

In the Phase III HIMALAYA trial [1], the combination of durvalumab (an anti-programmed cell death ligand-1 antibody) and tremelimumab (an anti-cytotoxic T-lymphocyte-associated protein 4 [CTLA-4] antibody) demonstrated an overall survival (OS) benefit over sorafenib in patients with advanced hepatocellular carcinoma (HCC). Based on these positive results, the combination of durvalumab and tremelimumab (Dur/Tre) is now recommended as a first-line systemic therapy for advanced HCC, as is atezolizumab plus bevacizumab (Atz/Bev) [2,3]. However, no reports have detailed Dur/Tre’s outcomes in actual clinical practice, and whether Dur/Tre will have the same efficacy and safety profile as in clinical trials has remained unclear.

Measuring concentrations of tumor markers for HCC, namely alpha-fetoprotein (AFP) and des-gamma-carboxyprotein (DCP), is less invasive and more common than tumor biopsy or imaging, and is widely used not only for diagnosing HCC, but also as an adjunctive diagnosis to determine treatment efficacy for HCC [4,5]. Several studies have reported associations between changes in AFP and DCP and treatment response and prognosis following treatment with molecularly targeted agents (MTAs) such as sorafenib [6,7,8] and lenvatinib [9,10,11] and in combined immunotherapy with Atz/Bev [12,13,14,15]. During the initial phase of using Dur/Tre at our institution, several cases were encountered in which patients with significant decreases in AFP and DCP early after the initiation of Dur/Tre showed good antitumor responses, whereas patients showing rapid increases in these markers showed poor antitumor responses. In the HIMALAYA study, changes in tumor markers during Dur/Tre were not reported in detail, and the relationship between changes in these tumor markers and the therapeutic effects of Dur/Tre have remained unclear.

In this study, we investigated changes in AFP, DCP, and the lens culinaris agglutinin-reactive fraction of alpha-fetoprotein (AFP-L3) in the early period after initiating Dur/Tre. We also evaluated the correlation between changes in these tumor markers and antitumor response to Dur/Tre. Finally, we also analyzed factors contributing to progression-free survival (PFS).

## 2. Materials and Methods

### 2.1. Patients

Between March 2023 and May 2024, 40 consecutive patients with advanced unresectable HCC started Dur/Tre therapy at our institution. Eligibility criteria included the following: (1) HCC diagnosis by imaging or biopsy, (2) Barcelona Clinic Liver Cancer (BCLC) stage C or B ineligible for surgery or locoregional therapy, (3) Eastern Cooperative Oncology Group performance status (ECOG-PS) of 0 or 1, and (4) Child–Pugh score of 7 or less. In addition, three patients were included: one with multinodular fused BCLC A and a maximum tumor diameter of 100 mm and two with Child–Pugh scores of 8 and 9 due to large tumor volume despite chronic hepatitis. All 40 patients were included in this study for retrospective evaluation of treatment outcomes.

### 2.2. Dur/Tre Treatment, Evaluation of Adverse Events, and Changes in Liver Function

All patients received durvalumab 1500 mg and tremelimumab 300 mg intravenously on the first administration. Thereafter, durvalumab 1500 mg was administered every 4 weeks. The treatment line refers to the sequence of systemic therapy with MTA or immune checkpoint inhibitors (ICIs). Adverse events (AEs) were evaluated according to the Common Terminology Criteria for Adverse Events (CT-CAE), 5th edition [16]. In the event of a drug-related AE, durvalumab was temporarily interrupted until symptoms improved to grade 1 or 2, according to guidelines provided by the manufacturer. Dur/Tre treatment was continued until a potentially fatal AE occurred or the tumor progressed clinically. To assess changes in liver function, albumin–bilirubin (ALBI) scores [17] were examined at baseline and at weeks 1, 2, 4, and 8 after Dur/Tre initiation.

### 2.3. Determination of Antitumor Response

Evaluation of antitumor response to Dur/Tre was performed according to the Response Evaluation Criteria in Solid Tumors version 1.1 (RECIST 1.1) [18] and modified RECIST (mRECIST) [19]. Dynamic computed tomography (CT) was performed at baseline, 8 weeks after initiation of Dur/Tre, and every 4–12 weeks thereafter according to a predefined schedule.

### 2.4. Evaluation of Changes in AFP, DCP, and AFP-L3

Serum AFP and DCP were measured as tumor markers for HCC at baseline and after 2, 4, and 8 weeks of Dur/Tre. AFP-L3 was measured at baseline and after 4 and 8 weeks of Dur/Tre. For each patient, the baseline concentration of each tumor marker was set to 1, and the ratio of each tumor marker to baseline was calculated at weeks 2, 4, and 8 of treatment. Analysis of tumor marker changes was performed in patients with AFP ≥ 10 ng/mL, DCP ≥ 40 mAU/mL, and AFP-L3 ≥ 0.5%, excluding those in the normal range of tumor markers at baseline.

### 2.5. Statistical Analysis

Statistical analysis was performed using Easy R (EZR) version 1.29 (Saitama Medical Center, Jichi Medical University) [20]. Statistical analysis was performed using Fisher’s direct probability test, the Mann–Whitney U test, Wilcoxon rank-sum test, Friedman test, Bonferroni method, etc., as appropriate. Cutoff values were determined using the receiver operating characteristic (ROC) curve and area under the curve (AUC). PFS was measured as the time from the start of Dur/Tre treatment to date of progression by RECIST 1.1 or date of death or last hospital visit. Overall survival (OS) was measured as the time from the start of Dur/Tre treatment to death or last hospital visit. PFS and OS were evaluated using the Kaplan–Meier method, and differences in survival were evaluated by the log-rank test. The Cox proportional hazards model was used to analyze factors contributing to PFS. For all analyses, the significance level was set at less than 0.05.

## 3. Results

### 3.1. Patient Characteristics at Baseline

Table 1 shows the baseline characteristics of the 40 HCC patients enrolled in this study at the start of Dur/Tre. Median age was 75 years, 33 patients were men, and 25 had non-viral HCC. The treatment line of Dur/Tre was first-line in 18 patients; 33 had ECOG-PS 0; 20 had a Child–Pugh score of 5; and 17 had BCLC stage C HCC. Median levels of AFP, DCP, and AFP-L3 at baseline were 108 ng/mL (range: 1.3–31,676 ng/mL), 603 mAU/mL (range: 10–162,000 mAU/mL), and 16.6% (range: <0.5–88.8%), respectively. The median neutrophil-to-lymphocyte ratio (NLR) level at baseline was 2.66 (range: 1.03–10.95). The median observation period was 7.6 months (range: 1.5–16.0 months).

### 3.2. Antitumor Response at 8 Weeks after Dur/Tre Initiation According to RECIST 1.1 and mRECIST

Antitumor responses at 8 weeks after Dur/Tre initiation according to RECIST 1.1 (8W-RECIST 1.1) were complete response (CR) in 0 patients, partial response (PR) in 10 patients, stable disease (SD) in 13 patients, progressive disease (PD) in 16 patients, and not evaluated (NE) in 1 patient (Table 2). The CR rate (CRR), objective response rate (ORR), and disease control rate (DCR) were 0%, 25.0%, and 57.5%, respectively. According to mRECIST at 8 weeks (8W-mRECIST) after Dur/Tre initiation, 3 patients had CR, 9 had PR, 11 had SD, 16 had PD, and 1 was NE, with a CRR of 7.5%, ORR of 30.0%, and DCR of 57.5%, respectively.

Baseline characteristics at Dur/Tre initiation, stratified by 8W-RECIST 1.1, are shown in Supplementary Materials Table S1. There were no significant differences in baseline factors between the 8W-OR and non-8W-OR groups or between the 8W-DC and non-8W-DC groups.

### 3.3. PFS and OS by 8W-RECIST 1.1

**Figure 1:**
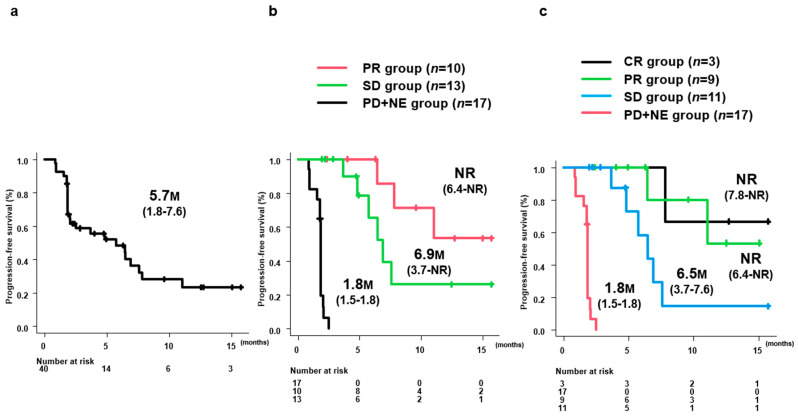
PFS in all patients and by 8W-RECIST 1.1. (**a**) Median PFS in all 40 patients was 5.7 months (95%CI: 1.8–7.6). (**b**) Median PFS by 8W-RECIST 1.1 was NR (95%CI: 6.4-NR) for PR group (*n* = 10), 6.9 months (95%CI: 3.7-NR) for SD group (*n* = 13), and 1.8 months (95%CI: 1.5–1.8) for PD+NE group (*n* = 17). (**c**) Median PFS by 8W-mRECIST was NR (95%CI: 7.8-NR) for CR group (*n* = 3), NR (95%CI: 6.4-NR) for PR group (*n* = 9), 6.5 months (95%CI: 3.7–7.6) for SD group (*n* = 11), and 1.8 months (95%CI: 1.5–1.8) for PD+NE group (*n* = 17). PFS, progression-free survival; M, months; 8W-RECIST 1.1, Response Evaluation Criteria in Solid Tumors version 1.1 at 8 weeks after initiation; mRECIST, modified RECIST; CR, complete response; PR, partial response; SD, stable disease; PD, progressive disease; NE, not evaluated; NR, not reached.

Median PFS in all 40 patients was 5.7 months (95% confidence interval [CI]: 1.8–7.6 months) (Figure 1a). Median PFS stratified by 8W-RECIST 1.1 was not reached (NR) (95%CI: 6.4–NR) in the PR group (*n* = 10), 6.9 months (95%CI: 3.7–NR) in the SD group (*n* = 13) and 1.8 months (95%CI: 1.5–1.8 months) in the PD+NE group (*n* = 17) (Figure 1b). Median PFS stratified by 8W-mRECIST was NR (95%CI: 7.8–NR) in the CR group (*n* = 3), NR (95%CI: 6.4–NR) in the PR group (*n* = 9), 6.5 months (95%CI: 3.7–7.6 months) in the SD group (*n* = 11), and 1.8 months (95%CI: 1.5–1.8 months) in the PD+NE group (*n* = 17) (Figure 1c).

**Figure 2:**
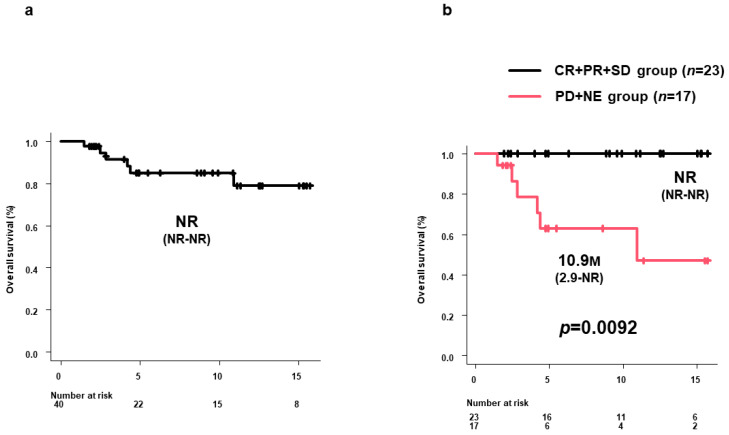
OS in all patients and by 8W-RECIST 1.1. (**a**) Median OS in all 40 patients was NR (95%CI: NR-NR). (**b**) Median OS in the CR+PR+SD group was NR (95%CI: NR-NR) but significantly longer than the 10.9 months (95%CI: 2.9 months-NR) in the PD+NE group (*p* = 0.0092). OS, overall survival; M, months; 8W-RECIST 1.1, Response Evaluation Criteria in Solid Tumors version 1.1 at 8 weeks after initiation; CR, complete response; PR, partial response; SD, stable disease; PD, progressive disease; NE, not evaluated; NR, not reached.

Median OS in all 40 patients was NR (95%CI: NR–NR) (Figure 2a). Median OS in the CR+PR+SD group was NR (95%CI: NR–NR), significantly longer than the 10.9 months (95%CI: 2.9 months—NR) in the PD+NE group (*p* = 0.0092). (Figure 2b)

### 3.4. AFP Ratios at 2, 4, and 8 Weeks after Dur/Tre Initiation, Stratified by 8W-RECIST 1.1

**Figure 3:**
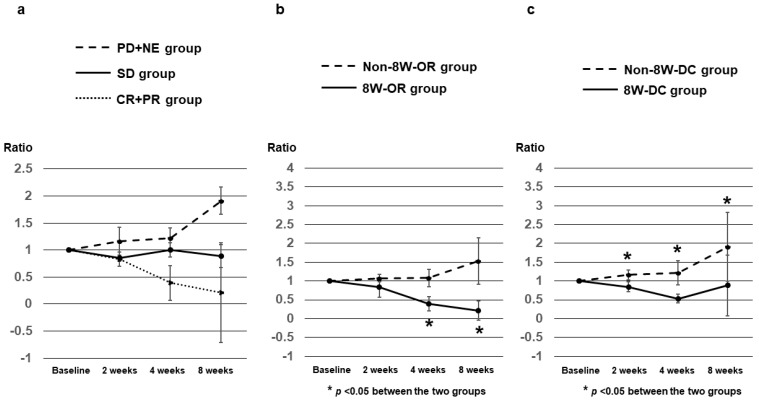
AFP ratios at 2, 4, and 8 weeks after Dur/Tre initiation, stratified by 8W-RECIST 1.1. (**a**) Median AFP ratios at 2, 4, and 8 weeks were 0.83, 0.39, and 0.21 in patients with CR+PR (*n* = 8); 0.85, 1.00, and 0.89 in patients with SD (*n* = 7); and 1.16, 1.22, and 1.91 in patients with PD+NE (*n* = 12), respectively; (**b**) Median AFP ratios in the CR+PR (8W-OR) group (*n* = 8) at weeks 4 and 8 were significantly lower than those in the SD+PD+NE (Non-8W-OR) group (*n* = 19). (**c**) Median AFP ratios in the CR+PR+SD (8W-DC) group (*n* = 15) at weeks 2, 4, and 8 were significantly lower than those in the PD+NE (Non-8W-DC) group (*n* = 12). AFP, alpha fetoprotein; 8W-RECIST 1.1, Response Evaluation Criteria in Solid Tumors version 1.1 at 8 weeks after initiation; CR, complete response; PR, partial response; SD, stable disease; PD, progressive disease; NE, not evaluated; OR, objective response; DC, disease control.

AFP ratios at 2, 4, and 8 weeks after Dur/Tre initiation, stratified according to 8W-RECIST 1.1, in 27 patients with a baseline AFP level ≥ 10 ng/mL are shown in Table 3. Median AFP ratios at 2, 4, and 8 weeks were 0.83, 0.39, and 0.21 in patients with CR+PR (*n* = 8); 0.85, 1.00, and 0.89 in patients with SD (*n* = 7); and 1.16, 1.22, and 1.91 in patients with PD+NE (*n* = 12), respectively (Figure 3a). Median AFP ratios in the CR+PR (8W-OR) group at weeks 4 and 8 were significantly lower than those in the SD+PD+NE (Non-8W-OR) group (*p* = 0.0068 and *p* = 0.0029, respectively) (Figure 3b). Median AFP ratios in the CR+PR+SD (8W-DC) group at weeks 2, 4, and 8 were significantly lower than those in the PD+NE (Non-8W-DC) group (*p* = 0.0127, *p* = 0.0006 and *p* = 0.0015, respectively) (Figure 3c).

ROC curves were plotted using 8W-OR and 8W-DC as the state variables and AFP ratios at weeks 2 and 4 as the test variables. For 8W-OR, the AUCs at weeks 2 and 4 were 0.64 and 0.84, with an optimal week 4 cutoff of 0.53 (75% sensitivity, 89.5% specificity). For 8W-DC, the AUCs at weeks 2 and 4 were 0.78 and 0.89, with an optimal week 4 cutoff of 1.01 (86.7% sensitivity, 91.7% specificity).

Actual AFP levels at 0, 2, 4, and 8 weeks after Dur/Tre initiation, stratified according to 8W-RECIST 1.1, are shown in Supplementary Materials Table S2. Median actual AFP levels in the 8W-DC group at weeks 4 and 8 were significantly lower than those in the Non-8W-DC group (*p* = 0.0128 and *p* = 0.0063, respectively).

### 3.5. DCP Ratios at 2, 4, and 8 Weeks after Dur/Tre Initiation, Stratified by 8W-RECIST 1.1

**Figure 4:**
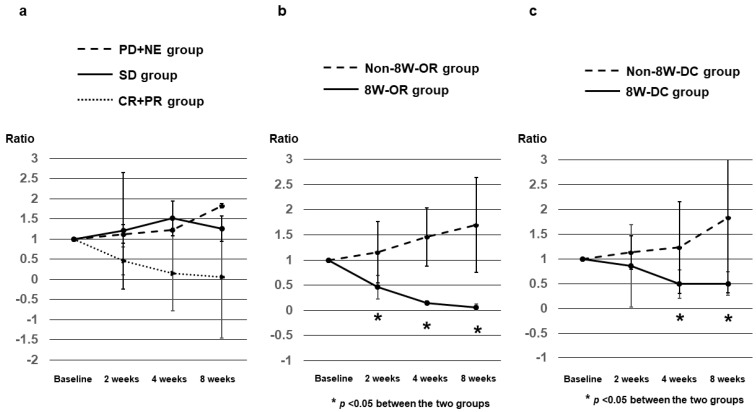
DCP ratios at 2, 4, and 8 weeks after Dur/Tre initiation, stratified by 8W-RECIST 1.1. (**a**) Median DCP ratios at 2, 4, and 8 weeks were 0.46, 0.15, and 0.06 in patients with CR+PR (*n* = 10); 1.21, 1.52, and 1.26 in patients with SD (*n* = 11); and 1.13, 1.23, and 1.83 in patients with PD+NE (*n* = 16), respectively. (**b**) Median DCP ratios in the 8W-OR group (*n* = 10) at weeks 2, 4, and 8 were significantly lower than those in the Non-8W-OR group (*n* = 27). (**c**) Median DCP ratios in the 8W-DC group (*n* = 21) at weeks 4 and 8 were significantly lower than those in the Non-8W-DC group (*n* = 16). DCP, des-γ-carboxy prothrombin; 8W-RECIST 1.1, Response Evaluation Criteria in Solid Tumors version 1.1 at 8 weeks after initiation; CR, complete response; PR, partial response; SD, stable disease; PD, progressive disease; NE, not evaluated; OR, objective response; DC, disease control.

DCP ratios at 2, 4, and 8 weeks after Dur/Tre initiation, stratified according to 8W-RECIST 1.1, in 37 patients with baseline DCP level ≥ 40 mAU/mL are shown in Table 4. Median DCP ratios at 2, 4, and 8 weeks were 0.46, 0.15, and 0.06 in patients with CR+PR (*n* = 10); 1.21, 1.52, and 1.26 in patients with SD (*n* = 11); and 1.13, 1.23, and 1.83 in patients with PD+NE (*n* = 16), respectively (Figure 4a). Median DCP ratios in the 8W-OR group at weeks 2, 4 and 8 were significantly lower than those in the Non-8W-OR group (*p* = 0.0049, *p* < 0.0001, and *p* < 0.0001, respectively) (Figure 4b). Median DCP ratios in the 8W-DC group at weeks 4 and 8 were significantly lower than those in the Non-8W-DC group *p* = 0.0215 and *p* = 0.0032, respectively) (Figure 4c).

ROC curves were plotted using 8W-OR and 8W-DC as the state variables and DCP ratios at weeks 2 and 4 as the test variables. For 8W-OR, the AUCs at weeks 2 and 4 were 0.84 and 0.98, with an optimal week 4 cutoff of 0.48 (100% sensitivity, 92.6% specificity). For 8W-DC, the AUCs at weeks 2 and 4 were 0.63 and 0.72, with an optimal week 4 cutoff of 0.73 (61.9% sensitivity, 81.2% specificity).

Actual DCP levels at 0, 2, 4, and 8 weeks after Dur/Tre initiation, stratified according to 8W-RECIST 1.1, are shown in Supplementary Materials Table S3. Median actual DCP levels in the 8W-OR group at weeks 4 and 8 were significantly lower than those in the Non-8W-OR group (*p* = 0.0167 and *p* = 0.0001, respectively). Median actual DCP level in the 8W-DC group at week 8 was significantly lower than that in the Non-8W-DC group (*p* = 0.0048).

### 3.6. AFP-L3 Ratios at 4 and 8 Weeks after Dur/Tre Initiation, Stratified by 8W-RECIST 1.1

**Figure 5:**
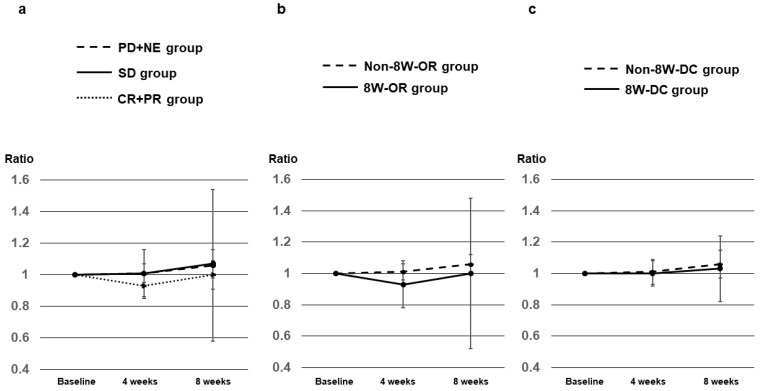
AFP-L3 ratios at 4 and 8 weeks after Dur/Tre initiation, stratified by 8W-RECIST 1.1. (**a**) Median AFP-L3 ratios at 4 and 8 weeks were 0.93 and 1.00 in patients with CR+PR (*n* = 9); 1.01 and 1.07 in patients with SD (*n* = 11); and 1.01 and 1.06 in patients with PD+NE (*n* = 13), respectively. (**b**) No significant differences in median AFP-L3 ratio were seen between the 8W-OR and Non-8W-PR groups. (**c**) No significant differences in median AFP-L3 ratio were seen between the Non-8W-DC and 8W-DC groups. AFP-L3, lens culinaris agglutinin-reactive fraction of alpha-fetoprotein; 8W-RECIST 1.1, Response Evaluation Criteria in Solid Tumors version 1.1 at 8 weeks after initiation; CR, complete response; PR, partial response; SD, stable disease; PD, progressive disease; NE, not evaluated; OR, objective response; DC, disease control.

AFP-L3 ratios at 4 and 8 weeks after Dur/Tre initiation, stratified according to 8W-RECIST 1.1, in 33 patients with a baseline AFP-L3 level ≥ 0.5% are shown in Table 5. Median AFP-L3 ratios at 4 and 8 weeks were 0.93 and 1.00 in 9 patients with CR+PR; 1.01 and 1.07 in 11 patients with SD; and 1.01 and 1.06 in 13 patients with PD+NE, respectively (Figure 5a). No significant differences in median AFP-L3 ratio were evident between the 8W-OR and Non-8W-OR groups and between the 8W-DC and Non-8W-DC groups (Figure 5b,c).

ROC curves were plotted using 8W-OR and 8W-DC as state variables and the AFP-L3 ratio at week 4 as the test variable. The AUC was 0.60 for 8W-OR and 0.51 for 8W-DC, both indicating low correlation.

Actual AFP-L3 levels at 0, 2, 4, and 8 weeks after Dur/Tre initiation, stratified according to 8W-RECIST 1.1, are shown in Supplementary Materials Table S4. The median actual AFP-L3 level in the 8W-DC group at week 8 was significantly lower than that in the Non-8W-DC group (*p* = 0.0390).

### 3.7. Prognostic Factors at Start of Dur/Tre Associated with Good PFS

Table 6 shows prognostic factors at the start of Dur/Tre associated with good PFS. Multivariate analysis showed that first-line treatment (hazard ratio [HR] = 0.374, 95%CI, 0.156–0.896; *p* = 0.0274) and NLR ≤ 3.00 (HR = 0.433; 95%CI, 0.188–0.996; *p* = 0.0490) were significant and independent predictors of good PPS.

### 3.8. PFS by Treatment Line and NLR

**Figure 6:**
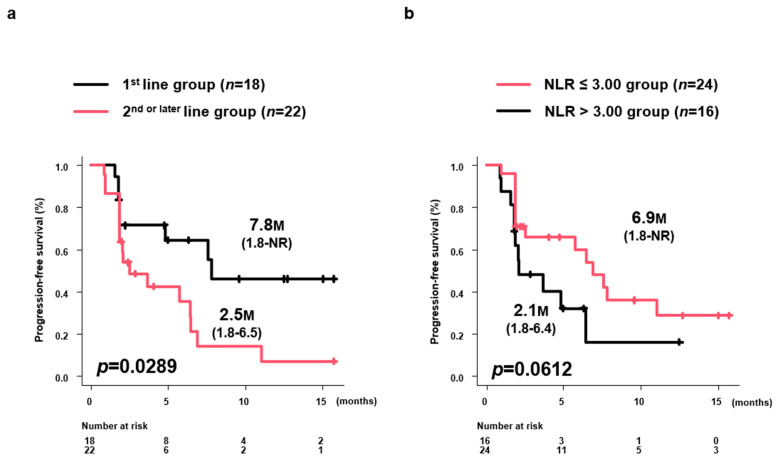
PFS by treatment line and NLR level. (**a**) Median PFS in the 1st-line group was 7.8 months (95%CI: 1.8 months-NR) but was significantly longer than the 2.5 months (95%CI: 1.8–6.5 months) in the 2nd-line or later group (*p* = 0.0289). (**b**) The median PFS in the NLR ≤ 3.00 group was 6.9 months (1.8 months-NR), which tended to be longer than the 2.1 months (1.8–6.4 months) in the NLR > 3.00 group (*p* = 0.0612). PFS, progression-free survival; M, months; NR, not reached; NLR, neutrophil-to-lymphocyte ratio.

Median PFS in the first-line group was 7.8 months (1.8 months–NR), significantly longer than the 2.5 months (1.8–6.5 months) in the second-line or later group (*p* = 0.0289) (Figure 6a). The median PFS in the NLR ≤ 3.00 group was 6.9 months (1.8 months-NR), which tended to be longer than the 2.1 months (1.8–6.4 months) in the NLR > 3.00 group (*p* = 0.0612) (Figure 6b).

### 3.9. Safety and Changes in Liver Function

Table 7 shows the incidence of AEs occurring within 8 weeks of Dur/Tre initiation. The most common AEs in all patients were fever, diarrhea, anorexia, general fatigue, and pruritus. Twenty-five percent of patients (*n* = 10) experienced grade 3 or higher AEs, with diarrhea being the most common, in five patients (12.5%). Six patients (15.0%) required steroids as treatment for immune-related AEs. This included three patients with grade 3 enteritis, two patients with grade 2 skin rashes, and one patient with grade 3 elevated aspartate aminotransferase. The median duration of treatment with Dur/Tre was 6.2 months (95% CI: 1.9–8.4).

**Figure 7:**
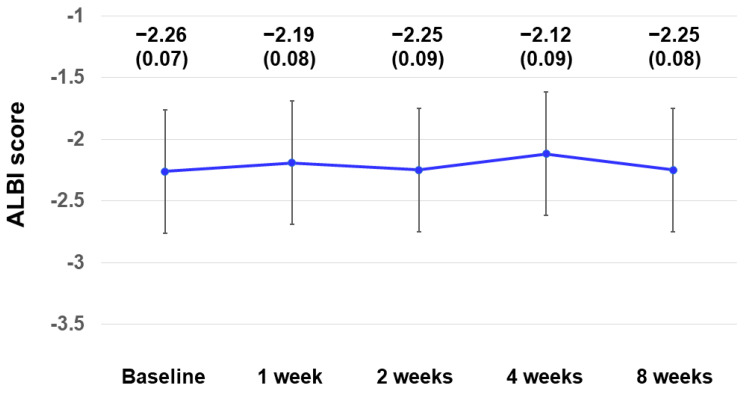
Changes in ALBI scores within 8 weeks in 40 patients. ALBI scores (median ± standard error) at baseline and weeks 1, 2, 4, and 8 were −2.26 ± 0.07, −2.19 ± 0.08, −2.25 ± 0.09, −2.12 ± 0.09, and −2.25 ± 0.08, respectively. ALBI, albumin–bilirubin.

Changes in ALBI scores within 8 weeks were assessed in 40 patients (Figure 7). The ALBI scores (median ± standard error (SE)) at baseline, weeks 1, 2, 4, and 8 were −2.26 ± 0.07, −2.19 ± 0.08, −2.25 ± 0.09, −2.12 ± 0.09, and −2.25 ± 0.08, respectively. There was no worsening of ALBI scores within 8 weeks.

### 3.10. Post-Progression Therapy

**Figure 8:**
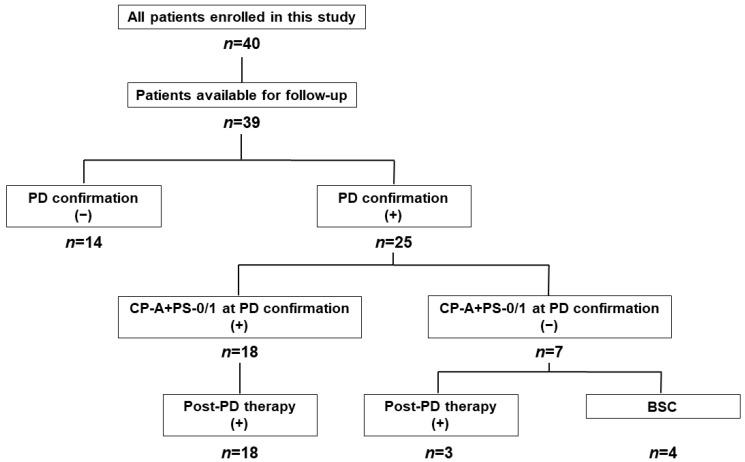
Flowchart on post-PD therapy. For patients with both Child–Pugh classification A and ECOG-PS 0 or 1 at time of PD confirmation, all 18 (100%) were able to progress to post-PD therapy, whereas only 3 of the remaining 7 patients (42.9%) were able to progress to post-PD therapy. PD, progressive disease; ECOG, Eastern Cooperative Oncology Group; PS, performance status; BSC, best supportive care.

In 39 patients, excluding one patient who was transferred to another hospital within 8 weeks and whose post-transfer status was unknown, post-progression therapy (post-PD therapy) was investigated (Figure 8). At cutoff, 25 patients were determined to have PD; 21 patients (84.0%) were switched to post-PD therapy. At the time of PD, eighteen patients (72.0%) met both Child–Pugh A and ECOG-PS 0 or 1 (CP-A+PS-0/1 group), and the remaining seven patients (non-CP-A+PS-0/1 group) did not meet both or either. In the CP-A+PS-0/1 group, all eighteen patients (100%) were able to switch to post-PD therapy (lenvatinib in six patients, Dur/Tre alone in five patients, cabozantinib in four patients, Dur/Tre+transarterial chemoembolization in two patients, and Atz/Bev in one patient), while only three patients (42.9%) in the non-CP-A+PS-0/1 group were able to switch to post-PD therapy (lenvatinib in two patients and Dur/Tre alone in one patient).

## 4. Discussion

This is the first study to focus on the correlation between early changes in tumor marker levels and antitumor response after Dur/Tre initiation in patients with advanced HCC in clinical practice. In particular, we found that early changes in AFP and DCP at 4 weeks after Dur/Tre initiation were both significantly associated with OR and DC according to RECIST 1.1 at 8 weeks. Factors at the start of Dur/Tre that were associated with good PFS were 1st-line Dur/Tre treatment and NLR ≤ 3.00.

Regarding the antitumor response of Dur/Tre according to RECIST 1.1, the HIMALAYA study reported an ORR of 20.1% and a DCR of 60.1% [1]. In the present study, antitumor response according to RECIST 1.1 at 8 weeks was similar, with an 8W-ORR of 25.0% and an 8W-DCR of 57.5%. An updated analysis of the HIMALAYA trial reported a 3-year survival rate of 44.6% and a 4-year survival rate of 36.2% for patients who achieved DC with the best response (60.1%) [21]. Dur/Tre therapy is called the STRIDE regimen, and the anti-CTLA-4 antibody tremelimumab is administered only once at the first time. In the course of systemic therapy for HCC, an initial single dose of a CTLA-4 inhibitor can provide long-term prognosis for some patients. Although the follow-up period in the current study was too short to draw conclusions, no deaths were seen among patients who achieved DC (CR+PR+SD group) with Dur/Tre treatment, and if DC can be achieved as in this clinical trial, good prognosis can be expected.

Several reports have examined the relationship between changes in AFP levels and antitumor response and prognosis in patients with advanced HCC treated with MTA and Atz/Bev [6,7,8,9,10,11,12,13,14,15]. Patients with decreased AFP levels during treatment reportedly achieved better antitumor response and prognosis, whereas patients with increased AFP levels showed worse antitumor response and prognosis. In the current study, the median AFP ratio was significantly lower in the 8W-OR group than in the non-8W-OR group at 4 and 8 weeks after starting Dur/Tre treatment. On the other hand, the median AFP ratio of the non-8W-DC group was significantly higher than that of the 8W-DC group at weeks 2, 4, and 8. The optimal cutoff values for the AFP ratio at week 4 predicting 8W-OR and 8W-DC were 0.53 and 1.01, respectively. These results suggest that in Dur/Tre therapy, as in other previously reported systemic therapy regimens, changes in AFP in the early treatment period may provide a useful predictor of antitumor response for both responsive cases and PD cases.

Several studies have also evaluated the relationship between changes in DCP after treatment initiation and antitumor efficacy and prognosis. Reports on MTA treatment have shown that, unlike AFP, early changes in DCP are not a useful predictor of antitumor efficacy or prognosis [6,22]. This is because some responders and SD patients who are considered to have achieved an antitumor effect also have elevated DCP, indistinguishable from the elevated DCP in PD. Tumor hypoxia due to anti-angiogenic therapy may lead to increased DCP production by the tumor itself [23]. In our own study, even with Atz/Bev treatment, early changes in DCP were not associated with antitumor efficacy [12]. In the present study, median DCP ratios among 8W-OR patients at weeks 2, 4, and 8 were significantly lower than those of non-8W-OR patients. DCP ratios at weeks 4 and 8 in the non-8W-DC group were also significantly higher than those in the 8W-DC group, suggesting that Dur/Tre treatment, unlike Atz/Bev treatment, does not involve VEGF inhibition and no mechanism may be needed for producing DCP from within the tumor due to hypoxia from inhibited angiogenesis. The optimal cutoff values for the DCP ratio at week 4 predicting 8W-OR and 8W-DC were 0.48 and 0.73, respectively. Therefore, in Dur/Tre treatment, changes in DCP and AFP changes may be associated with antitumor effects (response and PD cases).

Few reports have examined the relationship between changes in AFP-L3 and the efficacy of systemic therapy [13]. In the present study, AFP-L3 was unchanged in most patients after 4 weeks of treatment. After 8 weeks, a trend was seen toward a decrease in the 8W-OR group compared to the non-8W-OR group. With the advent of ICI therapy, including Atz/Bev, a small number of patients have been able to achieve clinical CR. In these patients, AFP-L3 eventually declines to normal levels. If a subsequent decrease in AFP-L3 is obtained, a favorable antitumor response can therefore be expected. However, based on the results of this study, AFP-L3, unlike AFP and DCP, may not offer a useful early predictor of antitumor response.

Combining multiple tumor markers or integrating markers with age and tumor factors has been reported as a useful indicator for predicting antitumor efficacy and prognosis [24,25,26,27]. Therefore, in Dur/Tre treatment, the use of a combination of these factors may be a more effective biomarker for predicting treatment outcome.

In this study, significant predictive factors at Dur/Tre initiation associated with good PFS were first-line Dur/Tre treatment and NLR ≤ 3.00. Although the number of cases in this study was small and the ability to draw conclusions is limited, the results suggest that, at least when Dur/Tre is introduced as a first-line treatment, we could expect similar results to those achieved in the HIMALAYA study. As for NLR, low NLR levels have been reported as predictive of good antitumor efficacy and favorable OS in ICI therapies, including Atz/Bev therapy for HCC [28,29,30,31,32,33]. Dur/Tre therapy is a combination of only two immune checkpoint inhibitors and, as with ICI therapy for other cancers, low NLR levels may provide a useful biomarker. In the present study, no correlation was found between baseline actual tumor marker levels and PFS, 8W-OR, and 8W-DCR (Table 6, Supplementary Materials Tables S1–S4).

ALBI scores were virtually unchanged within 8 weeks and no decrease in hepatic function was seen. At the time PD was confirmed by RECIST 1.1, 72.0% of patients met the requirements of Child–Pugh A and ECOG-PS 0 or 1 scores for recommended transition to subsequent systemic therapy. This is a higher percentage than has been reported for other MTA treatments [34,35,36]. In fact, all these patients were able to switch to post-PD therapy. Even if PD was found, favorable conditions (good hepatic function and general condition) could be expected to allow subsequent treatments to be fully effective and prolong prognosis. The median age of patients in this study was 75 years, 10 years older than in the HIMALAYA study (65 years), but the incidence of AEs was similar to that of the HIMALAYA study. Dur/Tre was considered a well-tolerated therapy in real clinical practice when AEs were detected and treated early.

This study showed several limitations. First, this was a retrospective, non-randomized study. Second, the sample size was small and the duration of follow-up was short. Additional studies with a larger number of patients in independent cohorts are thus needed to corroborate the findings of the current study. This study included only patients with baseline tumor marker levels above the upper limit of normal. Thus, it did not evaluate the relationship between tumor marker changes and antitumor efficacy in patients with normal levels, limiting the conclusions.

## 5. Conclusions

In conclusion, early changes in tumor markers after the initiation of Dur/Tre treatment were associated with antitumor response. In particular, changes in AFP and DCP at week 4 may be useful biomarkers for early prediction of both response and PD following initiation of Dur/Tre treatment.

## Figures and Tables

**Table 1:** Baseline characteristics at initiation of durvalumab plus tremelimumab.

Patient Characteristics	*n* = 40
Age, years; median (range)	75 (40–91)
Sex, male/female	33/7
Etiology, HBV/HCV/non-viral	6/9/25
Treatment line, 1st/2nd/3rd/4th/5th	18/7/11/2/2
ECOG-PS, 0/1	33/7
Child–Pugh score, 5/6/7/8/9	20/13/5/1/1
mALBI grade, 1/2a/2b/3	12/9/19/1
BCLC stage, A/B/C	1/22/17
Intrahepatic tumor number, <4/≥4	11/29
Maximum intrahepatic tumor size, <50 mm/≥50 mm	26/14
Portal vein tumor thrombosis, 0/1/2/3/4	30/0/5/4/1
Extrahepatic metastasis, −/+	27/13
AFP level, ng/mL; median (range)	108 (1.3–31,676)
DCP level, mAU/mL; median (range)	603 (10–162,000)
AFP-L3 level, %; median (range)	16.6 (<0.5–88.8)
NLR; median (range)	2.66 (1.03–10.95)
Observation period, months; median (range)	7.6 (1.5–16.0)

HBV, hepatitis B virus; HCV, hepatitis C virus; non-viral, non-HBV and non-HCV; ECOG, Eastern Cooperative Oncology Group; PS, performance status; mALBI, modified albumin–bilirubin; BCLC, Barcelona Clinic Liver Cancer; AFP, alpha-fetoprotein; DCP, des-γ-carboxy prothrombin; AFP-L3, lens culinaris agglutinin-reactive fraction of alpha-fetoprotein; NLR, neutrophil-to-lymphocyte ratio.

**Table 2:** Antitumor response according to RECIST 1.1 and mRECIST at 8 weeks after initiating durvalumab plus tremelimumab (*n* = 40).

	CR *n* (%)	PR *n* (%)	SD *n* (%)	PD *n* (%)	NE *n* (%)	CRR	ORR	DCR
8W-RECIST 1.1	0 (0)	10 (25.0)	13 (32.5)	16 (40.0)	1 (2.5)	0%	25.0%	57.5%
8W-mRECIST	3 (7.5)	9 (22.5)	11 (27.5)	16 (40.0)	1 (2.5)	7.5%	30.0%	57.5%

W, weeks; RECIST 1.1, Response Evaluation Criteria in Solid Tumors version 1.1; mRECIST, modified RECIST; CR, complete response; PR, partial response; SD, stable disease; PD, progressive disease; NE, not evaluated; CRR, complete response rate; ORR, objective response rate; DCR, disease control rate.

**Table 3:** AFP ratio at 2, 4, and 8 weeks after initiating durvalumab and tremelimumab stratified by 8W-RECIST 1.1 (*n* = 27).

8W-RECIST 1.1	AFP Ratios, Median (SE)					*p* Value	
	CR+PR (8W-OR) *n* = 8	SD *n* = 7	PD+NE (Non-8W-DC) *n* = 12	CR+PR+SD (8W-DC) *n* = 15	SD+PD+NE (Non-8W-OR) *n* = 19	8W-OR vs. Non-8W-OR	8W-DC vs. Non-8W-DC
At 2W	0.83 (0.26)	0.85 (0.06)	1.16 (0.13)	0.84 (0.13)	1.07 (0.10)	0.3033	0.0127
At 4W	0.39 (0.19)	1.00 (0.13)	1.22 (0.32)	0.53 (0.12)	1.08 (0.23)	0.0068	0.0006
At 8W	0.21 (0.25)	0.89 (0.21)	1.91 (0.92)	0.88 (0.18)	1.53 (0.61)	0.0029	0.0015

AFP, alpha fetoprotein, 8W-RECIST 1.1, Response Evaluation Criteria in Solid Tumors version 1.1 at 8 weeks after initiation; SE, standard error; CR, complete response; PR, partial response; SD, stable disease; PD, progressive disease; NE, not evaluated; OR, objective response; DC, disease control; W, weeks.

**Table 4:** DCP ratio at 2, 4, and 8 weeks after initiating durvalumab and tremelimumab stratified by 8W-RECIST 1.1 (*n* = 37).

8W-RECIST 1.1	DCP Ratios, Median (SE)					*p* Value	
	CR+PR (8W-OR) *n* = 10	SD *n* = 11	PD+NE (Non-8W-DC) *n* = 16	CR+PR+SD (8W-DC) *n* = 21	SD+PD+NE (Non-8W-OR) *n* = 27	8W-OR vs. Non-8W-OR	8W-DC vs. Non-8W-DC
At 2W	0.46 (0.23)	1.21 (1.45)	1.13 (0.34)	0.86 (0.83)	1.15 (0.61)	0.0049	0.2057
At 4W	0.15 (0.04)	1.52 (0.43)	1.23 (0.93)	0.49 (0.29)	1.46 (0.58)	<0.0001	0.0215
At 8W	0.06 (0.06)	1.26 (0.31)	1.83 (1.51)	0.50 (0.24)	1.70 (0.94)	<0.0001	0.0032

DCP, des-γ-carboxy prothrombin; 8W-RECIST 1.1, Response Evaluation Criteria in Solid Tumors version 1.1 at 8 weeks after initiation; SE, standard error; CR, complete response; PR, partial response; SD, stable disease; PD, progressive disease; NE, not evaluated; OR, objective response; DC, disease control; W, weeks.

**Table 5:** AFP-L3 ratio at 2, 4, and 8 weeks after initiating durvalumab and tremelimumab stratified by 8W-RECIST 1.1 (*n* = 33).

8W-RECIST 1.1	AFP-L3 Ratios, Median (SE)					*p* Value	
	CR+PR (8W-OR) *n* = 9	SD *n* = 11	PD+NE (Non-8W-DC) *n* = 13	CR+PR+SD (8W-DC) *n* = 20	SD+PD+NE (Non-8W-OR) *n* = 24	8W-OR vs. Non-8W-OR	8W-DC vs. Non-8W-DC
At 4W	0.93 (0.15)	1.01 (0.06)	1.01 (0.08)	1.00 (0.08)	1.01 (0.05)	0.3675	0.9388
At 8W	1.00 (0.48)	1.07 (0.09)	1.06 (0.09)	1.03 (0.21)	1.06 (0.06)	0.4188	0.6060

AFP-L3, lens culinaris agglutinin-reactive fraction of alpha-fetoprotein; 8W-RECIST 1.1, Response Evaluation Criteria in Solid Tumors version 1.1 at 8 weeks after initiation; SE, standard error; CR, complete response; PR, partial response; SD, stable disease; PD, progressive disease; NE, not evaluated; OR, objective response; DC, disease control; W, weeks.

**Table 6:** Uni- and multivariate survival analyses of factors at initiation of durvalumab plus tremelimumab associated with good PFS.

Factors	Univariate Analysis		Multivariate Analysis	
	HR (95%CI)	*p* Value	HR (95%CI)	*p* Value
Age (<75 years)	0.841 (0.381–1.857)	0.6685		
Sex (female)	1.588 (0.591–4.271)	0.3594		
Etiology (HBV or HCV)	1.390 (0.619–3.117)	0.4248		
Treatment line (1st)	0.404 (0.171–0.958)	0.0395	0.374 (0.156–0.896)	0.0274
ECOG-PS (0)	0.536 (0.199–1.441)	0.2164		
Child–Pugh score (5)	0.728 (0.330–1.606)	0.4320		
BCLC stage (A or B)	0.642 (0.292–1.411)	0.2697		
Number of intrahepatic tumors (≥4)	1.216 (0.452–3.269)	0.6986		
Maximum size of intrahepatic tumors (≥50 mm)	0.851 (0.365–1.983)	0.7088		
Portal vein tumor thrombosis (+)	1.466 (0.624–3.444)	0.3804		
Extrahepatic metastasis (+)	0.528 (0.236–1.181)	0.1200		
AFP level (≥100 ng/mL)	1.470 (0.660–3.277)	0.3458		
DCP level (≥400 mAU/mL)	1.105 (0.502–2.432)	0.8039		
AFP-L3 level (≥10.0%)	1.044 (0.458–2.380)	0.9178		
NLR (≤3.00)	0.477 (0.211–2.347)	0.0757	0.433 (0.188–0.996)	0.0490

PFS, progression-free survival; HR, hazard ratio; CI, confidence interval; HBV, hepatitis B virus; HCV, hepatitis C virus; ECOG, Eastern Cooperative Oncology Group; PS, performance status; BCLC, Barcelona Clinic Liver Cancer; AFP, alpha-fetoprotein; DCP, des-γ-carboxy prothrombin; AFP-L3, lens culinaris agglutinin-reactive fraction of alpha-fetoprotein; NLR, neutrophil-to-lymphocyte ratio.

**Table 7:** Adverse events within 8 weeks of initiating durvalumab plus tremelimumab (*n* = 40).

Adverse Event	Any Grade *n* (%)	Grade 1/2 *n* (%)	Grade 3/4 *n* (%)
Fever	8 (20.0)	8 (20.0)	0
Diarrhea	7 (17.5)	2 (5.0)	5 (12.5)
Anorexia	7 (17.5)	6 (15.0)	1 (2.5)
General fatigue	7 (17.5)	7 (17.5)	0
Pruritus	7 (17.5)	7 (17.5)	0
Elevated aspartate aminotransferase	3 (7.5)	1 (2.5)	2 (5.0)
Skin rash	3 (7.5)	3 (7.5)	0
Deterioration of liver function	2 (5.0)	0	2 (5.0)
Reduced adrenal function	1 (2.5)	1 (2.5)	0
Hypothyroidism	1 (2.5)	1 (2.5)	0

## Data Availability

All data generated or analyzed in this study are included in this article. Please direct any inquiries to the corresponding author.

## Supplementary Materials

The following supporting information can be downloaded at: https://www.mdpi.com/article/10.3390/curroncol31080315/s1, Table S1. Baseline characteristics at initiation of durvalumab plus tremelimumab, stratified by 8W-RECIST 1.1; Table S2. Actual AFP level at 0, 2, 4, and 8 weeks after initiating durvalumab and tremelimumab, stratified by 8W-RECIST 1.1 (*n* = 27).; Table S3. Actual DCP level at 0, 2, 4, and 8 weeks after initiating durvalumab and tremelimumab, stratified by 8W-RECIST 1.1 (*n* = 37).; Table S4. Actual AFP-L3 level at 0, 2, 4, and 8 weeks after initiating durvalumab and tremelimumab, stratified by 8W-RECIST 1.1 (*n* = 33).
