# Supplementary material for: Early Changes in Alpha-Fetoprotein and Des-γ-Carboxy Prothrombin Are Useful Predictors of Antitumor Response to Durvalumab Plus Tremelimumab Therapy for Advanced Hepatocellular Carcinoma
Source: Curr Oncol. 2024 Jul 26;31(8):4225–40. doi: 10.3390/curroncol31080315 (PMC11353012; doi:10.3390/curroncol31080315)
Supplement: Supplementary file 1 [file curroncol-31-00315-s001.zip › curroncol-3101078-supplementary.pdf]

Supplementary Table S1. Baseline characteristics at initiation of durvalumab plus tremelimumab, stratified by 8W-RECIST 1.1.

8W-RECIST 1.1	Patient characteristics			*p* value	
	CR+PR (8W-OR) n=10	SD n=13	PD+NE (Non-8W-DC) n=17	8W-OR vs Non-8W-OR	8W-DC vs Non-8W-DC
Age, <75 years / ≥75 years	6/4	5/8	8/9	0.4727	1.0000
Sex, male/ female	9/1	10/3	14/3	0.6560	1.0000
Etiology, viral/ non-viral	3/7	5/8	7/10	0.7148	0.7486
Treatment line, 1st/ 2nd or later	5/5	7/6	6/11	0.7307	0.3476
ECOG-PS, 0/ 1	10/0	11/2	12/5	0.1612	0.1134
Child–Pugh score, 5/ 6 or more	7/3	5/8	8/9	0.2733	1.0000
BCLC stage, A or B/ C	4/6	11/2	8/9	0.2743	0.3368
Intrahepatic tumor number, <4/ ≥4	1/9	4/9	6/11	0.2328	0.4774
Maximum intrahepatic tumor size, <50 mm/ ≥50 mm	5/5	9/4	12/5	0.2779	0.7385
Portal vein tumor thrombosis, -/ +	6/4	13/0	11/6	0.2323	0.2743
Extrahepatic metastasis, -/ +	7/3	11/2	9/8	1.0000	0.1709
AFP level, <100 ng/mL / ≥100 ng/mL	3/7	9/4	7/10	0.2812	0.5378
DCP level, <400 mAU/mL / ≥400 mAU/mL	6/4	6/7	7/10	1.0000	0.5378
AFP-L3 level, <10.0% / ≥10.0%	3/7	6/7	7/10	0.7110	1.0000
NLR; ≤3.00 / >3.00	8/2	8/5	8/9	0.2633	0.1991

8W-RECIST 1.1, Response Evaluation Criteria in Solid Tumors version 1.1 at 8 weeks after initiation; ECOG, Eastern Cooperative Oncology Group; PS, performance status; BCLC, Barcelona Clinic Liver Cancer; AFP, alpha-fetoprotein; DCP, des-γ-carboxy prothrombin; AFP-L3, lens culinaris agglutinin-reactive fraction of alpha-fetoprotein; NLR, neutrophil-to-lymphocyte ratio.

Supplementary Table S2. Actual AFP level at 0, 2, 4, and 8 weeks after initiating durvalumab and tremelimumab, stratified by 8W-RECIST 1.1 (n = 27).

8W-RECIST 1.1	Actual AFP levels (ng/mL), median (SE)					*p* value	
	CR+PR (8W-OR) n=8	SD n=7	PD+NE (Non-8W-DC) n=12	CR+PR+SD (8W-DC) n=15	SD+PD+NE (Non-8W-OR) n=19	8W-OR vs Non-8W-OR	8W-DC vs Non-8W-DC
**At 0W**	700 (1034)	102 (64)	1045 (2820)	307 (581)	307 (1853)	0.4572	0.1571
**At 2W**	451 (2699)	86.9 (53.7)	598 (3306)	175 (1374)	296.5 (2144)	0.7165	0.1124
**At 4W**	348.5 (1054)	103 (39.1)	1513.5 (3636)	111 (571)	277 (2440)	0.5954	0.0128
**At 8W**	381.6 (617)	97.9 (62.9)	1038.5 (5220)	97.9 (1338)	437 (3524)	0.2220	0.0063

AFP, alpha fetoprotein; 8W-RECIST 1.1, Response Evaluation Criteria in Solid Tumors version 1.1 at 8 weeks after initiation; SE, standard error; CR, complete response; PR, partial response; SD, stable disease; PD, progressive disease; NE, not evaluated; OR, objective response; DC, disease control; W, weeks.

Supplementary Table S3. Actual DCP level at 0, 2, 4, and 8 weeks after initiating durvalumab and tremelimumab, stratified by 8W-RECIST 1.1 (n = 37).

8W-RECIST 1.1	Actual DCP levels (mAU/mL), median (SE)					*p* value	
	CR+PR (8W-OR) n=10	SD n=11	PD+NE (Non-8W-DC) n=16	CR+PR+SD (8W-DC) n=21	SD+PD+NE (Non-8W-OR) n=27	8W-OR vs Non-8W-OR	8W-DC vs Non-8W-DC
At 0W	224 (17566)	811 (2172)	2347 (1859)	455 (8615)	1842 (1387)	0.6941	0.5197
At 2W	1975 (16205)	1608 (2174)	2530 (1890)	1608 (7314)	2403 (1400)	0.7687	0.7449
At 4W	32 (1265)	1204 (1894)	2959 (1997)	529 (1166)	2097 (1407)	0.0167	0.0754
At 8W	30	2180	4279	244	3061	0.0001	0.0048

	(78)	(2192)	(5011)	(1224)	(3167)		

DCP, des-γ-carboxy prothrombin; 8W-RECIST 1.1, Response Evaluation Criteria in Solid Tumors version 1.1 at 8 weeks after initiation; SE, standard error; CR, complete response; PR, partial response; SD, stable disease; PD, progressive disease; NE, not evaluated; OR, objective response; DC, disease control; W, weeks.

Supplementary Table S4. Actual AFP-L3 level at 0, 2, 4, and 8 weeks after initiating durvalumab and tremelimumab, stratified by 8W-RECIST 1.1 ($n = 33$).

8W-RECIST 1.1	Actual AFP-L3 levels (%), median (SE)					*p* value	
	CR+PR (8W-OR) n=9	SD n=11	PD+NE (Non-8W-DC) n=13	CR+PR+SD (8W-DC) n=20	SD+PD+NE (Non-8W-OR) n=24	8W-OR vs Non-8W-OR	8W-DC vs Non-8W-DC
**At 0W**	42.7 (11.1)	16.1 (4.2)	60.6 (8.7)	17.6 (5.9)	25.6 (5.9)	0.7160	0.0972
**At 4W**	19.1 (10.8)	15.1 (4.8)	60.8 (9.2)	15.9 (5.9)	29.8 (6.3)	0.9833	0.1202
**At 8W**	13.0 (9.4)	11.1 (5.0)	57.0 (9.3)	12.2 (5.0)	24.2 (6.2)	0.5713	0.0390

AFP-L3, lens culinaris agglutinin-reactive fraction of alpha-fetoprotein; 8W-RECIST 1.1, Response Evaluation Criteria in Solid Tumors version 1.1 at 8 weeks after initiation; SE, standard error; CR, complete response; PR, partial response; SD, stable disease; PD, progressive disease; NE, not evaluated; OR, objective response; DC, disease control; W, weeks.
